# Supplementary material for: Functional analysis of a hypomorphic allele shows that MMP14 catalytic activity is the prime determinant of the Winchester syndrome phenotype
Source: Hum Mol Genet. 2018 May 8;27(16):2775–88. doi: 10.1093/hmg/ddy168 (PMC6077784; doi:10.1093/hmg/ddy168)
Supplement: Supplementary Data [file ddy168_supp.zip › MMP14 paper supplemental data Revision submitted.docx]

**Supplemental materials and methods**

*Microarray based molecular karyotyping for Copy Number Variant (CNV) detection*

Copy Number Variant (CNV) analysis was carried out on the patients’ gDNA using the Affymetrix high resolution CytoScan® High Density (HD) platform (Affymetrix Inc., Santa Clara, CA, USA), following the manufacturer's protocol. The CytoScan microarray provides genome wide coverage with >2.6 million combined SNP and copy number probes. Genotype calls were generated from scanned data files using the Affymetrix GeneChip® Command Console Software, and subsequently analyzed for segment calling to detect copy number variants using the Affymetrix Chromosome Analysis Suite (CHAS®) software version 2.0. The patients’ data was analyzed against a control reference data set obtained on the same platform from 50 mixed-sex unaffected individuals. CNV breakpoints were mapped to UCSC hg19/NCBI.37 builds and Ensembl Release 69 positions.

*Microarray based identification of regions showing loss of heterozygosity (LoH)*

LoH mapping was carried out on the raw genotyping data in order to facilitate identification of candidate genes with a possible autosomal recessive heritability mode. The HomozygosityMapper tool was used as developed and made available for on-line use at the Cologne Center for Genomics and Institute for Genetics (Cologne University, Germany) by Seelow et al. following the author’s recommendations [S1].

*Whole exome sequencing*

Genomic DNA was isolated from peripheral blood leukocytes. Whole exome sequencing in the proband was outsourced to the Department of Clinical Genetics of Maastricht University Medical Center+. Briefly, the quality of the genomic DNA was determined for purity using Nanodrop (NanoDrop products, Wilmington, USA) ratio value 260/280nm of 1.91, tested for degradation with 0.8% standard Agarose gel and finally quantified for 4 µg starting material with PicoGreen on a Qubit fluorometer (Invitrogen, Bleiswijk, the Netherlands). The sample was additionally cleaned up after quantification with a DNA Clean & Concentrator^TM^ (ZymoResearch, BaseClear Diagnostics, Leiden, the Netherlands). The genomic DNA was sheared between 150-200 bp lengths with Covaris S2 (Covaris Inc., Massachusetts, USA). The exome was captured according to the SureSelect Human All Exon V4 plus UTR's (Agilent, Technologies, Amstelveen, the Netherlands) for Paired-End Illumina Sequencing platforms.

The exome kit included a targeted content of 71 Mb with 20,965 genes, 335,765 exons, designed according databases like CCDS (March 2011), RefSeq (March 2011), GENCODE (v6), miRBase (v17), TCGA (v6) and UCSC (March 2011). Exome enrichment was carried out using standard SureSelect protocol and quality steps were performed using the Bioanalyzer 2100 (Agilent). Fragments were end- repaired, ligated to specific adapters after addition of a 3'A and specifically amplified in a pre-PCR step for 6 cycles under standard conditions in a thermal cycler to yield a total amount of 500 ng product. Hybridization was performed for 72 h at 65°C. The specific hybridized exome targets was captured with biotin labelled probes, the binding of streptavidin beads (Dynabeads MyOne Streptavidin T1, Life Technologies AS, Oslo, Norway) was performed and additional multiple wash steps removed nonspecific targets. A post-PCR step for the enriched exome targets was performed for 12 cycles under standard conditions using a thermal cycler for single indexing. Afterwards a quality check was performed for the library size on a High Sensitivity DNA Kit and Bioanalyzer 2100 (Agilent) and additionally quantified with Qubit (Invitrogen) for equimolar pooling of libraries during sequencing. Clusters were generated using the cluster generation kit v3 for Paired-End sequencing on the cBot for program v8 according to the manufacturer’s protocol (Illumina, Eindhoven, the Netherlands). All clustered captured fragments were subsequently sequenced using 2 x 100 cycles v3 on Paired-End by sequencing by synthesis (SBS) on a HighSeq 2000 platform. Basecalling was done using Casava version 1.8.2 (Illumina). A total of 154,052,858 reads with an average length of 92 nucleotides were sequenced and 99.89% of these mapped reads were aligned to the human reference genome (UCSC hg19) using Burrows-Wheeler-Alignment tool (BWA) version 0.5.9. This resulted in an on-target average depth of 108x, already corrected for a percentage of 31.34% duplicate reads for this exome with a specificity of 79.83% reads on target. Single base variants were identified using the Genome Analysis Toolkit (GATK) version 2.2 and annotated using an in-house developed oracle based annotation database that matched variants to the Refgene and dbSNP137 hg19 tracks from the UCSC genome browser. All differences identified between the probands sequence and the reference sequence were listed and annotated in Microsoft® Excel®. Variant annotation included chromosomal position, genotype/zygosity, coverage, gene, predicted effect on the encoded protein and (if known) population frequency. The gene mutation identified by whole exome sequencing was verified by conventional Sanger sequencing. To this end, a 376-bp fragment spanning the relevant portion of the gene was amplified using primers MMP14F3 (5'-cttggcctttccccacattg-3') and MMP14R3 (5'-gccattttcctccccactcc-3') and sequenced using the BigDye Terminator v1.1 Cycle Sequencing kit (Applied Biosystems, Bleiswijk, The Netherlands) on an ABI 3730 DNA Analyser.

**Legends to supplemental figures and movies**

**Figure S1 – Two brothers with a novel homozygous *MMP14* missense mutation. A**, pedigree of our patients (arrows). The parents and sister were not affected. The parents were consanguineous (first cousins); the mother died of mammary carcinoma at age 40. **B**, homozygosity mapping identified seven loss-of-heterozygosity (LoH) regions (red). **C**, details of the seven identified LoH regions. **D**, the parents and sister were heterozygous, whereas both brothers were homozygous for a novel c.332G>A missense mutation in exon 4 of *MMP14*, identified by whole exome sequencing.

**Figure S2 – Endogenous MMP14 is expressed at lower levels by MRC5 cells compared to HT1080 cells, but localizes similarly. A**, anti-MMP14 (Millipore mab3328) immunoblot on whole cell protein extract (left panel) demonstrates low endogenous MMP14 levels in MRC5 cells compared to HT1080 cells. Anti-KU-80 (Cell Signaling Technology, 2180) immunoblot (middle panel) served as loading control. **B**, immunofluorescence microscopy images of MRC5 and HT1080 cells stained with an anti-MMP14 antibody (Abcam, Ab51074) demonstrate that endogenous MMP14 localizes almost identically in both cell types, namely at the cell surface and in a perinuclear aggregate. Scale bars equal 20 µm.

**Figure S3 – Exogenously expressed double-tagged MMP14 WT localizes similarly to endogenous MMP14 in MRC5 cells.** Immunofluorescence microscopy images of MRC5 cells stained with an anti-MMP14 antibody (Abcam, Ab51074) demonstrate that the double-tagged MMP14 WT fusion protein localizes in a similar fashion to endogenous MMP14. Cells stained for endogenous MMP14 expressed 3HA-EGFP as transfection control. Scale bars equal 20 µm.

**Figure S4 –** **The R111H mutation does not impair MMP14 cell surface localization, in contrast to mutations T17R, R92C and S466P.** Subcellular localization of WT and mutant 3HA-MMP14-EGFP fusion proteins exogenously expressed in MRC5 cells (same cells as shown in Fig. 1). The region of the left panels highlighted with a square is enlarged in the middle panels. Images were scaled with ImageJ using bilinear interpolation. Right panels show profile plots of the normalized pixel intensity of EGFP and F-actin corresponding to the region marked with the arrows in the middle panels. MMP14 WT-EGFP (i) and MMP14 R111H-EGFP (iv) are present at the cell surface where they colocalize with F-actin, as indicated by the overlapping white signal in merged IF images (left and middle panels) and coinciding peak intensity (right panels). The other mutant MMP14-EGFP fusion proteins and 3HA-EGFP control are absent from the cell surface; note the decline in EGFP intensity right before the F-actin peak intensity at the cell surface. Scale bars equal 20 µm.

**Figure S5 – Knockout of *mmp14a* and *mmp14b* in zebrafish.** **A**, schematic of *mmp14a* and *mmp14b* introns and exons (top, introns not drawn to scale) and protein domain structure (bottom). Two sequences (red solid lines) in exon 4 of either *mmp14a* or *mmp14b* were targeted for genomic editing by CRISPR/Cas9. Exon 4 of both genes encodes a major part of the catalytic domain. Frameshift mutation at exon 4 was predicted to lead to a premature stop codon, deleting the C-terminal functional domains including the Zn (red dot)-binding sites in the catalytic domain of any resulting protein. S, signal peptide; St, stalk region; T, transmembrane domain; I, cytoplasmic tail. **B & C**, mutant *mmp14a* and *mmp14b* mRNA undergo nonsense-mediated decay. Displayed are the mRNA expression levels for *mmp14a* (left) or *mmp14b* (right) of twenty 1-5 dpf offspring of WT fish (black bars) or either *mmp14a*^∆/∆^;*mmp14b*^+/+^ (B) or *mmp14a*^+/+^;*mmp14b*^∆/∆^ (C) fish intercrossed. Expression levels are normalized for β-actin expression and denoted relative to 1 dpf WT embryos (dotted horizontal line). Error bars represent SEM of technical triplicates. **D**, *in vitro* translation of mutant *mmp14a* and *mmp14b* mRNA does not result in full-length protein. Four expression vectors were generated encoding WT or mutant Mmp14a or Mmp14b with a C-terminal HA-tag from cDNA of WT or mutant zebrafish with the forward (Fw) and reverse (Rv) primers indicated (see also Table S2). Anti-HA (Cell Signaling Technology, 3724) immunoblot on whole cell protein extract of MRC5 cells expressing these constructs demonstrates a strong band at the expected height for the WT Mmp14a-HA (60 kDa) and WT Mmp14b-HA (71 kDa) fusion proteins. No bands are detected for the mutant forms, indicating the disruptive effect of the selected mutations.

**Figure S6 – Knockout of *mmp14a* and *mmp14b* does not affect larval craniofacial cartilage elements and vertebral mineralization rate. A**, schematic overview of the craniofacial cartilage elements in 5 dpf larvae, including their relative intensity when stained with Alcian blue and imaged as wholemount in ventral view, anterior to the left. Abbreviations: cb1-4, ceratobranchial 1-4; ch, ceratohyal; et, ethmoid; h, hyosymplectic; mk, Meckel’s cartilage; pf, pectoral fin; pq, palatoquadrate. **B**, the craniofacial cartilage elements in 5 dpf *mmp14a/b* KO larvae are qualitatively normal in size, shape and staining intensity compared to WT larvae. A minimum of 12 individuals per genotype was analyzed; representative images are shown. **C**, schematic overview of the Weberian (W), prehemal (ph), hemal (h) and caudal fin (cf) vertebrae, hypural complex (hc) and supranumeral rays (sr) with the sequence of mineralization waves (1-3); lateral view, anterior to the left. **D**, the number of calcified vertebral ossification centra does not differ between WT and *mmp14a/b* KO fish at 14 dpf. Note the large proportion of larvae that have not yet started mineralization of the vertebral column; this proportion does not differ significantly between the genotypes. Horizontal lines indicate mean ± SEM. A minimum of 39 individuals per genotype was analyzed. **E**, the average ossification score (number of mineralized elements as shown in C corrected for standard length; blue line) does not differ between WT and KO juveniles at 21 dpf. A minimum of 14 individuals per genotype was analyzed. The cartoons in panel A and C are adapted from Aceto et al. respectively Bensimon-Brito et al., under the terms of the Creative Commons Attribution License (URL: https://creativecommons.org/licenses/by/4.0/ and URL: https://creativecommons.org/ licenses/by/2.0/, respectively) [S2, S3].

**Figure S7 – Affected skull bones in *mmp14a/b* KO fish have altered collagen content yet normal collagen fibril organization.** Picrosirius red (PSR) staining of sagittal sections (anterior to the left except for the *mmp14a/b* KO section shown in panel E and F, which is rotated -45° (anterior at the bottom) for clearer comparison with the corresponding WT section) demonstrate an uneven staining (arrowheads) and cell clusters (asterisks) giving the frontal bones (A) of *mmp14a/b* KO fish a “threadbare” appearance compared to WT fish. The dentary (C) and supraoccipital bones (SOC, E) of *mmp14a/b* KO fish lack a collagen-rich cortex observed in WT fish. The SOC additionally contains cell-free regions (asterisks) and clusters of multinucleated cells (arrowheads). Polarized DIC imaging of PSR stained sections revealed no overt differences in birefringence, in frontal bones (B), maxilla (D) and SOC (F), indicating collagen deposition is unaffected by *mmp14a/b* KO. Scale bars equal 20 µm.

**Movie S1 – Tumbling movements in *mmp14a/b* KO fish prior to death.** Movie demonstrating swimming pattern of four *mmp14a/b* KO fish, three swimming normally and one (bottom right at the start of the movie) making tumbling movements, frequently observed in these mutant fish 1-2 days prior to their deaths.

**Supplemental tables**

Table S1 – Primers for cloning and mutagenesis of pQCXIB 3HA-MMP14-EGFP vectors.

| **MMP14 WT cDNA** | | | |
| --- | --- | --- | --- |
| Fw |  | 5’-TGAATTCCAATGTCTCCCGCCCCAAGA-3’ |  |
| Rv |  | 5’-AGATCTTGACCTTGTCCAGCAGGGAACGC-3’ |  |
|  |  |  |  |
| **SDM MMP14 T17R** | | | |
| Fw |  | 5’-CGTTGTCTCCTGCTCCCCCTGCTCAGGCTCGGCACCGCGCTCGCCTCCCTC-3’; |  |
| Rv |  | 5’-GAGGGAGGCGAGCGCGGTGCCGAGCCTGAGCAGGGGGAGCAGGAGACAAC  G-3’ |  |
|  |  |  |  |
| **SDM MMP14 R92C** | | | |
| Fw |  | 5’-CCATGAGGCGCCCCTGCTGTGGTGTTCCAG-3’ |  |
| Rv |  | 5’- CTGGAACACCACAGCAGGGGCGCCTCATGG-3’ |  |
|  |  |  |  |
| **SDM MMP14 R111H** | | | |
| Fw |  | 5’-AATGTTCGAAGGAAGCACTACGCCATCCAGGGTCTCAAATGG-3’ |  |
| Rv |  | 5-ACCCTGGATGGCGTAGTGCTTCCTTCGAACATTGGCCTTGAT-3’ |  |
|  |  |  |  |
| **SMD MMP14 S466P** | | | |
| Fw |  | 5’-GAGTCTCCCAGAGGGCCATTCATGGGCAGCGATGAAGTCT-3’ |  |
| Rv |  | 5’-CGCTGCCCATGAATGGCCCTCTGGGAGACTCAGGGATCCC-3’ |  |
|  |  |  |  |
| **Generation of 3HA-EGFP vector** | | | |
| Fw |  | 5’-CTCGAGCTCAAGCTTCGAATTCCAAAGATCCACCGGCCGGTAGCCACC-3’ |  |
| Rv |  | 5’-GGTGGCTACCGGCCGGTGGATCTTTGGAATTCGAAGCTTGAGCTCGAG-3’ |  |

F, forward; mut, mutation; or, orientation; R, reverse; SDM, site-directed mutagenesis. Altered codon is underlined in the primer sequences.

Table S2 – Primers used to generate *mmp14a/b* plasmids.

| **Name** |  | **Sequence** |  | **Tm** (˚C) |
| --- | --- | --- | --- | --- |
| *mmp14a* Fw |  | 5’-CCACCATGTTACCGAAACTGCAGACG-3’ |  | 56.0 |
| *mmp14a* Rv |  | 5’-TTAAGCGTAATCTGGAACATCGTATGGGTAAACCTTATC  GAGCAGAGAGCG-3’ |  | 58.0 |
| *mmp14b* Fw |  | 5’-CCACCATGATCTGGAGCGGGTTCA-3’ |  | 56.0 |
| *mmp14b* Rv |  | 5’-TTAAGCGTAATCTGGAACATCGTATGGGTAAACCTTGTC  CAGTAGGGAGC-3’ |  | 58.0 |

Fw, forward; Rv, reverse; Tm, melting temperature.

Table S3 – Primary antibodies used. IF, immunofluorescence; WB, western blot.

| **Raised against** |  | **Raised in** |  | **Clonality** |  | **Manufacturer** |  | **Product code** |  | **Concentration** | | |
| --- | --- | --- | --- | --- | --- | --- | --- | --- | --- | --- | --- | --- |
|  |  |  |  |  |  |  |  |  |  | **IF** |  | **WB** |
| β-actin |  | rabbit |  | poly |  | Cell Signaling |  | 4867 |  | - |  | 1:4,000 |
| GFP |  | mouse |  | duo |  | Roche |  | 11814460001 |  | - |  | 1:1,000 |
| HA |  | mouse |  | mono |  | Abcam |  | Ab16918 |  | 1:200 |  | - |
| HA |  | rabbit |  | mono |  | Cell Signaling |  | 3724 |  | - |  | 1:1,000 |
| KU-80 |  | rabbit |  | mono |  | Cell Signaling |  | 2180 |  | - |  | 1:1,000 |
| MMP14 |  | mouse |  | mono |  | Millipore |  | mab3328 |  | - |  | 1:1,000 |
| MMP14 |  | rabbit |  | mono |  | Abcam |  | Ab51074 |  | 1:100 |  | - |

Table S4 – Primers used for qPCR.

| **Name** |  | **Sequence** |  | **Binds to exon** |  | **Product** |  | **Tm**  (˚C) |
| --- | --- | --- | --- | --- | --- | --- | --- | --- |
| *mmp14a* Fw |  | 5’-CAAGGAAAAGTGGTTCTGGCGA-3’ |  | 6-7 |  | 186 bp |  | 58.6 |
| *mmp14a* Rv |  | 5’-CCTTCCTCCATTTTAGCCTCGTTG-3’ |  | 8 |  |  |  | 60.5 |
| *mmp14b* Fw |  | 5’-ACTCCATACCCCACACCGTA-3’ |  | 6 |  | 130 bp |  | 58.4 |
| *mmp14b* Rv |  | 5’-CACGCCAGAACCACTTACCCTTA-3’ |  | 6-7 |  |  |  | 60.5 |
| *β-actin* Fw |  | 5’-CGAGCAGGAGATGGGAACC-3’ |  | 1-2 |  | 100 bp |  | 60.4 |
| *β-actin* Rv |  | 5’-CAACGGAAACGCTCATTGC-3’ |  | 2-3 |  |  |  | 56.1 |

Fw, forward; Rv, reverse; Tm, melting temperature.

**Supplemental references**

S1. Seelow, D. and Schuelke, M. (2012) HomozygosityMapper2012--bridging the gap between homozygosity mapping and deep sequencing. *Nucleic Acids Res.*, **40**, W516-520.

S2. Aceto, J., Nourizadeh-Lillabadi, R., Maree, R., Dardenne, N., Jeanray, N., Wehenkel, L., Alestrom, P., van Loon, J.J. and Muller, M. (2015) Zebrafish Bone and General Physiology Are Differently Affected by Hormones or Changes in Gravity. *PLoS One*, **10**, e0126928.

S3. Bensimon-Brito, A., Cardeira, J., Cancela, M.L., Huysseune, A. and Witten, P.E. (2012) Distinct patterns of notochord mineralization in zebrafish coincide with the localization of Osteocalcin isoform 1 during early vertebral centra formation. *BMC Dev. Biol.*, **12**, 28.
