# Supplementary figures and images for: Functional analysis of a hypomorphic allele shows that MMP14 catalytic activity is the prime determinant of the Winchester syndrome phenotype
Source: Hum Mol Genet. 2018 May 8;27(16):2775–88. doi: 10.1093/hmg/ddy168 (PMC6077784; doi:10.1093/hmg/ddy168)

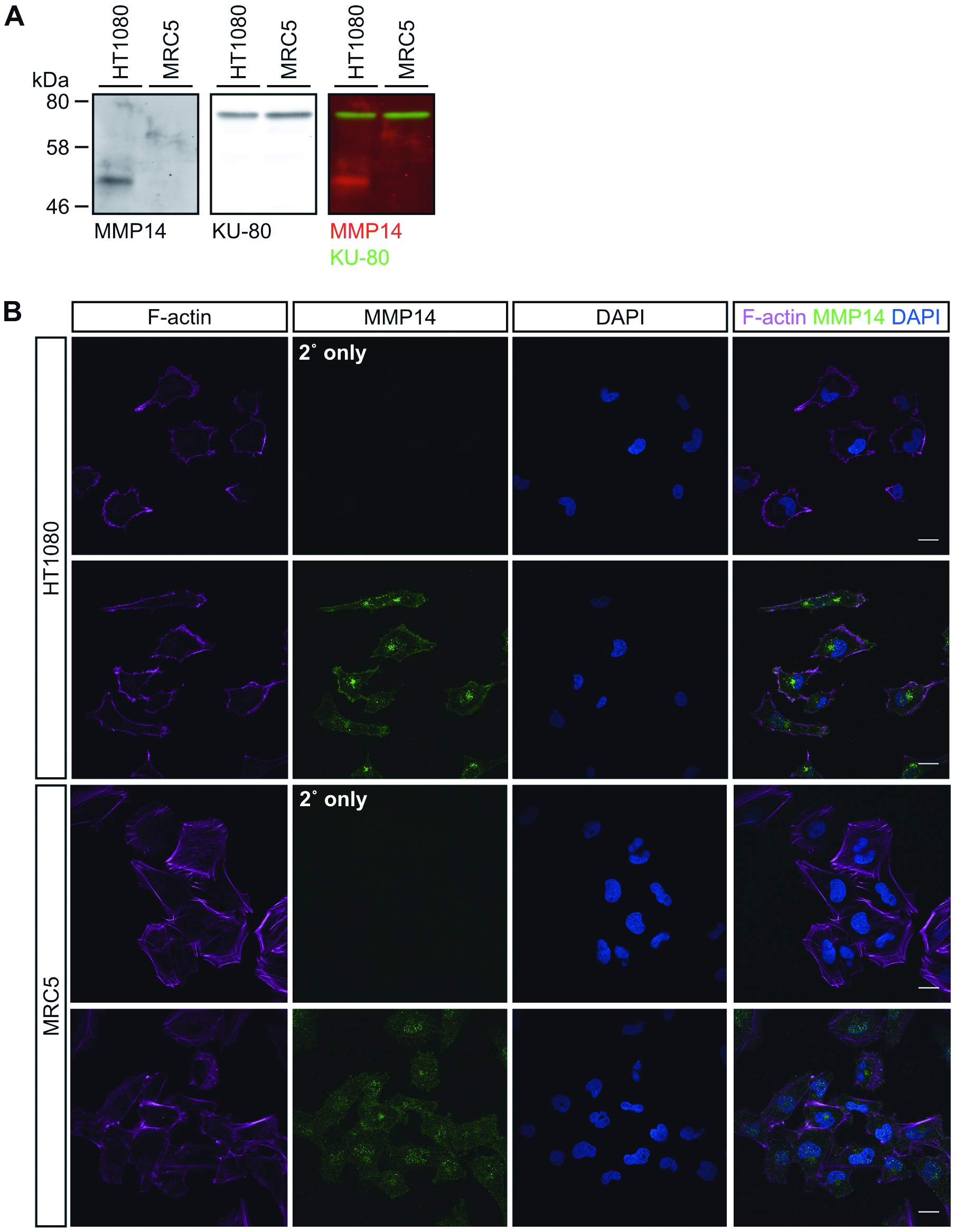

Supplement: Supplementary Data [file ddy168_supp.zip › Fig S2 Revision_HMG.tif]

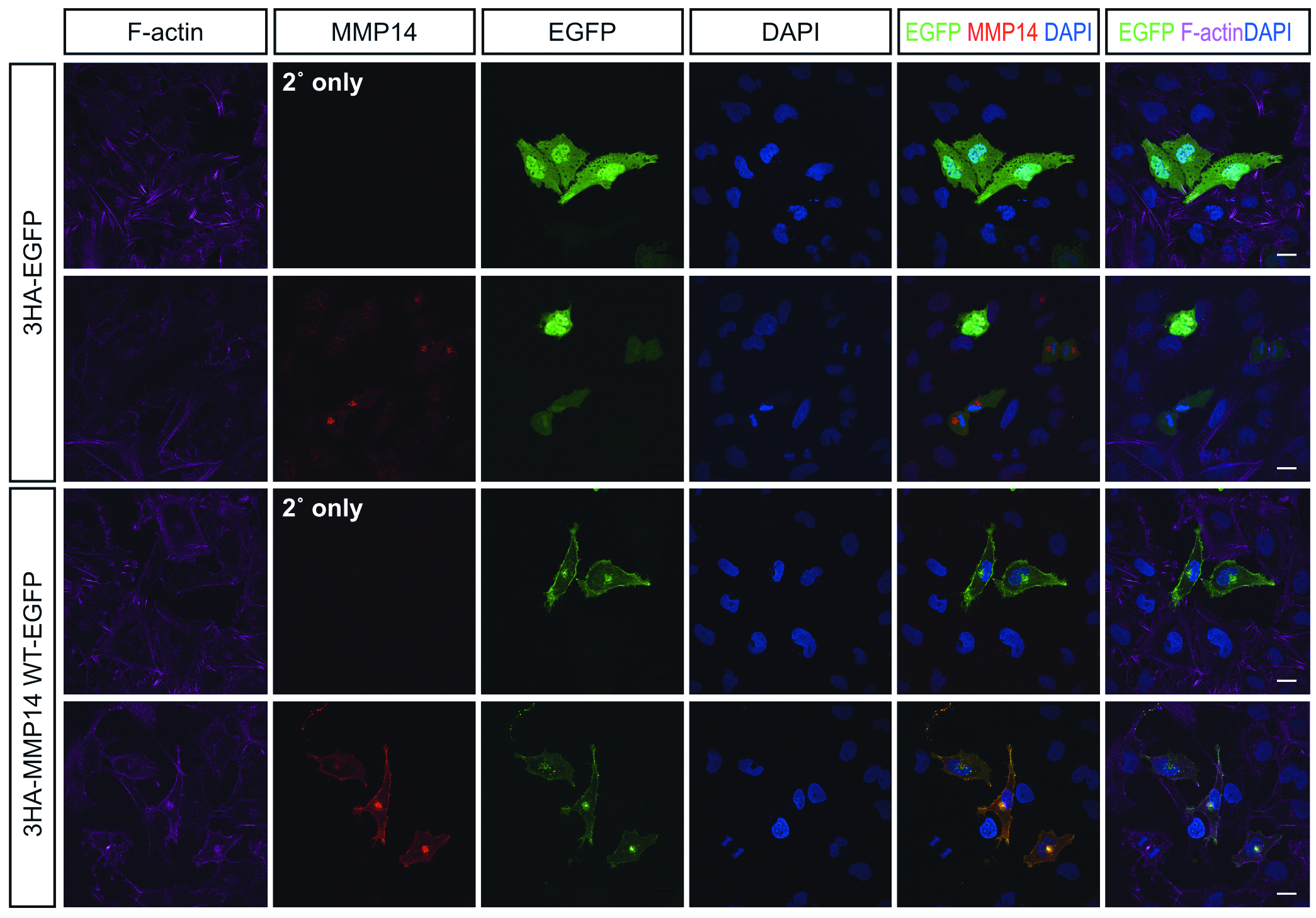

Supplement: Supplementary Data [file ddy168_supp.zip › Fig S3 Revision_HMG.tif]

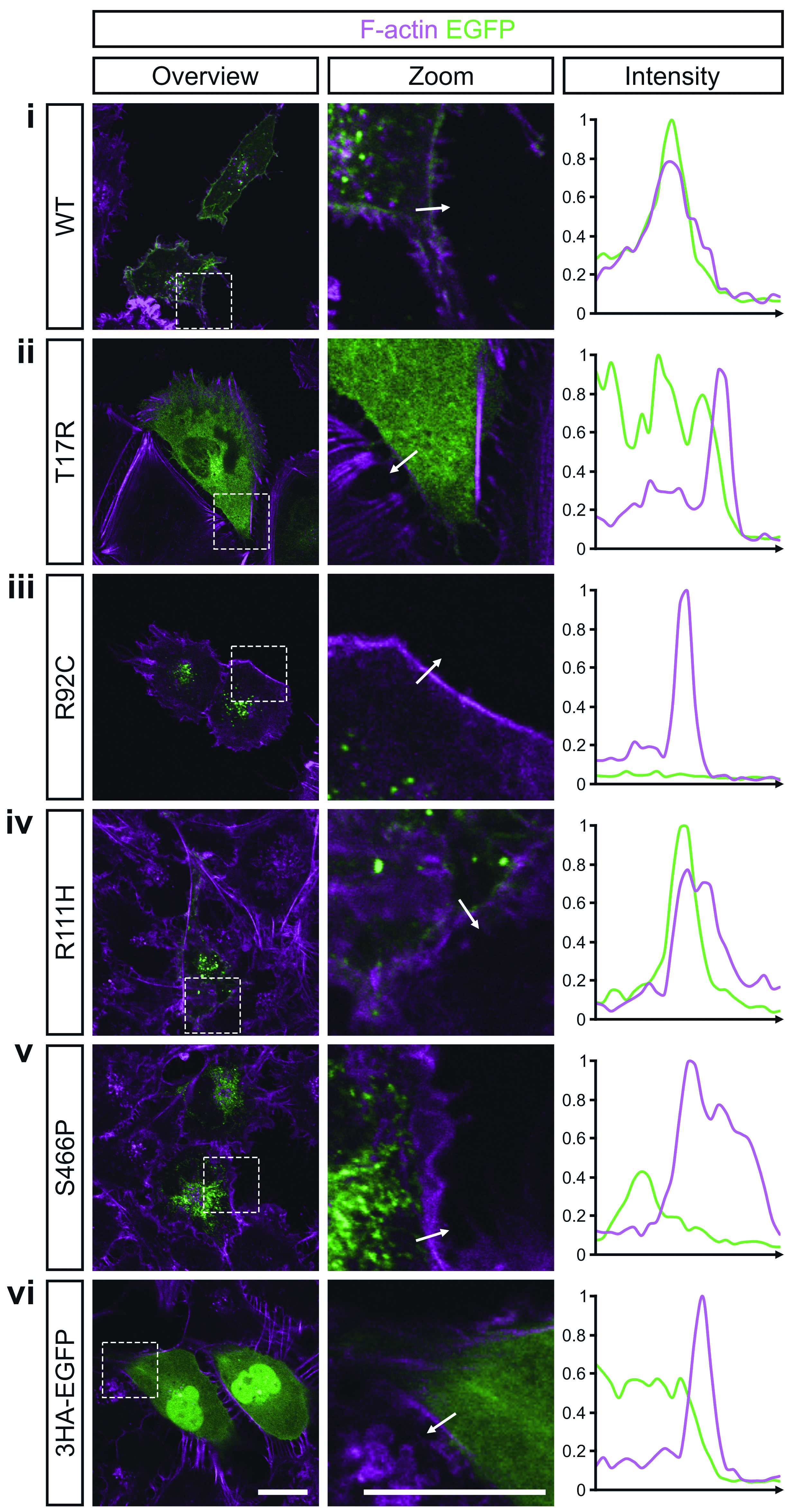

Supplement: Supplementary Data [file ddy168_supp.zip › Fig S4_Revision_HMG.tif]

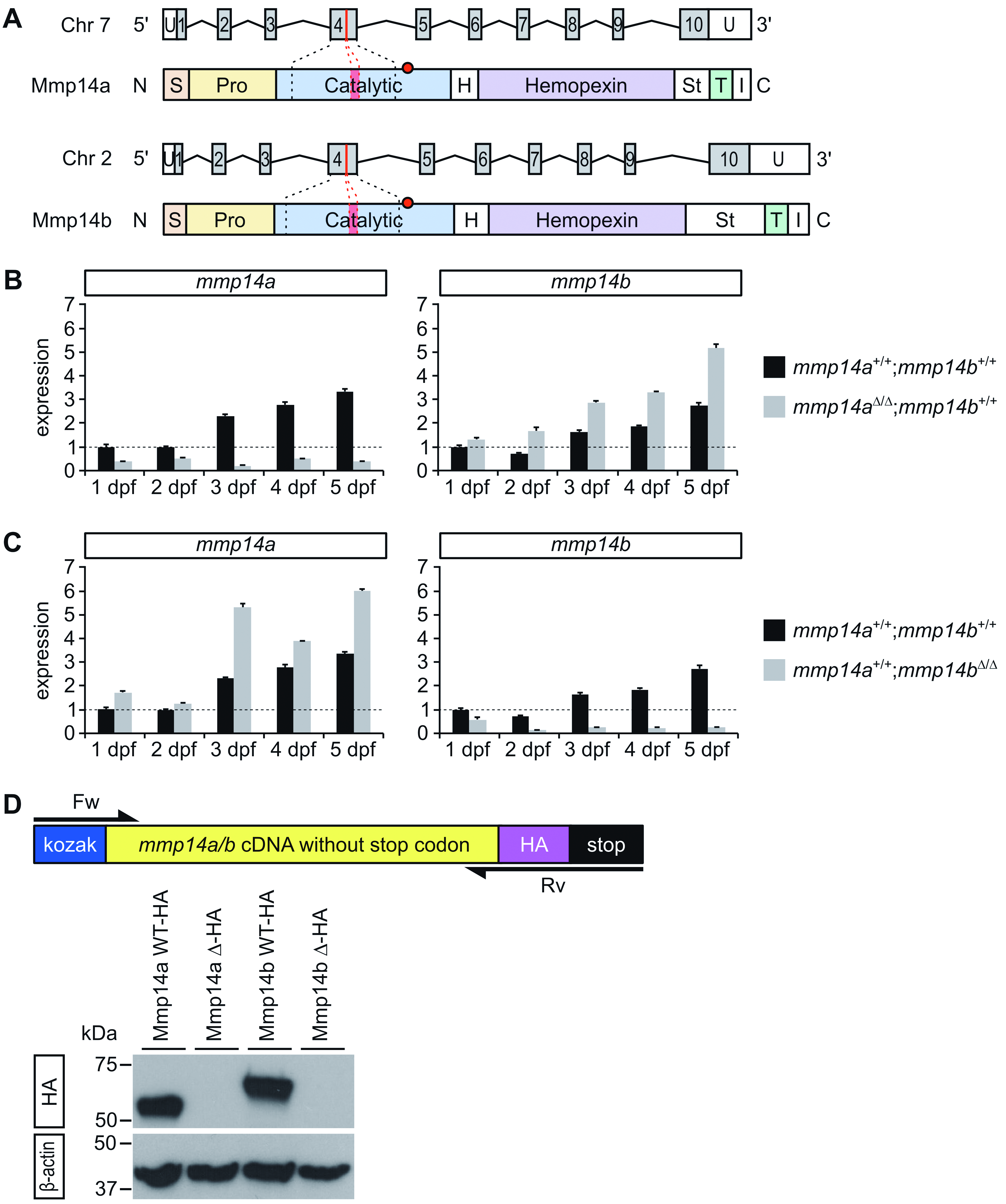

Supplement: Supplementary Data [file ddy168_supp.zip › Fig S5_Revision_HMG.tif]

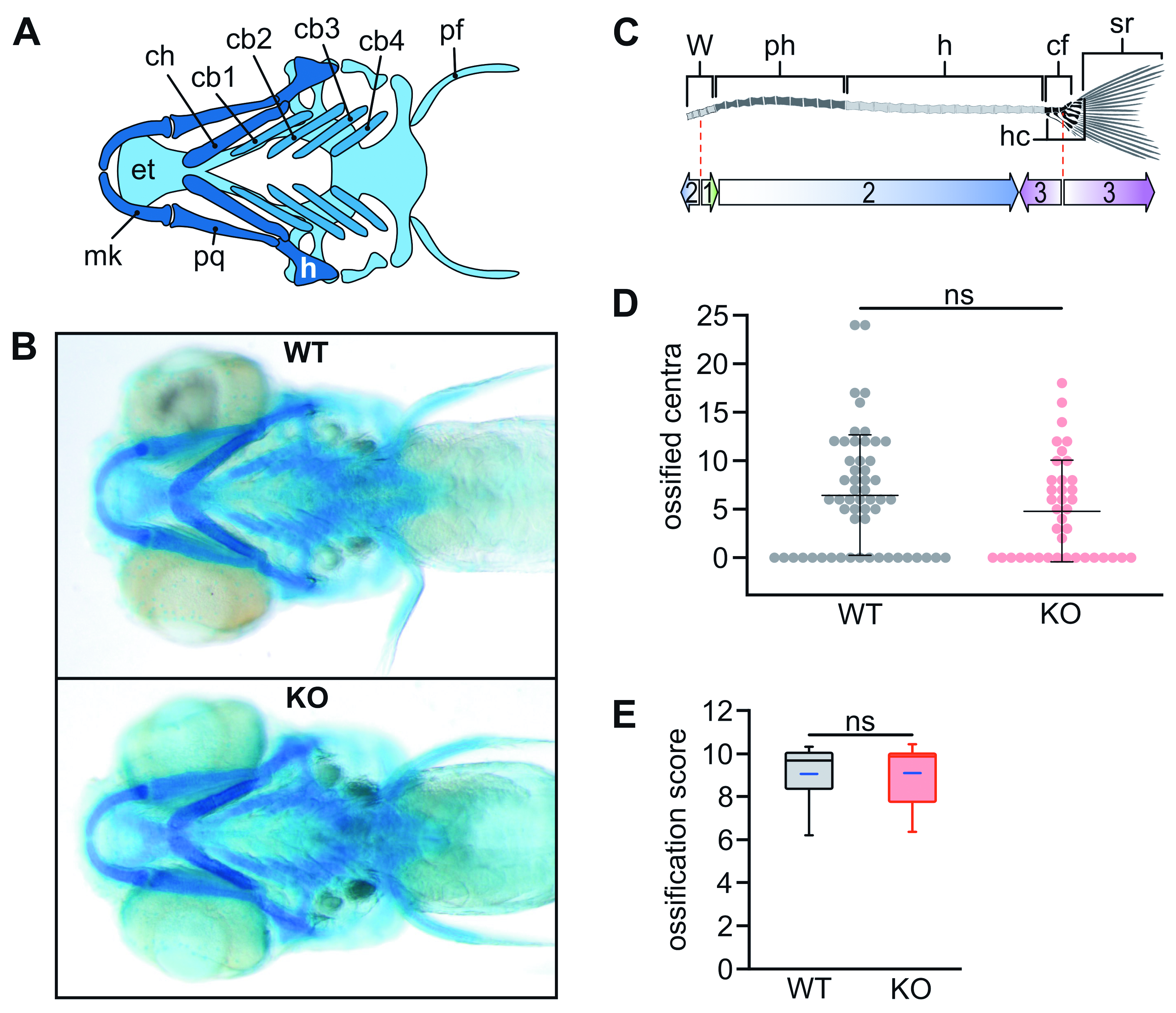

Supplement: Supplementary Data [file ddy168_supp.zip › Fig S6 Revision_HMG.tif]

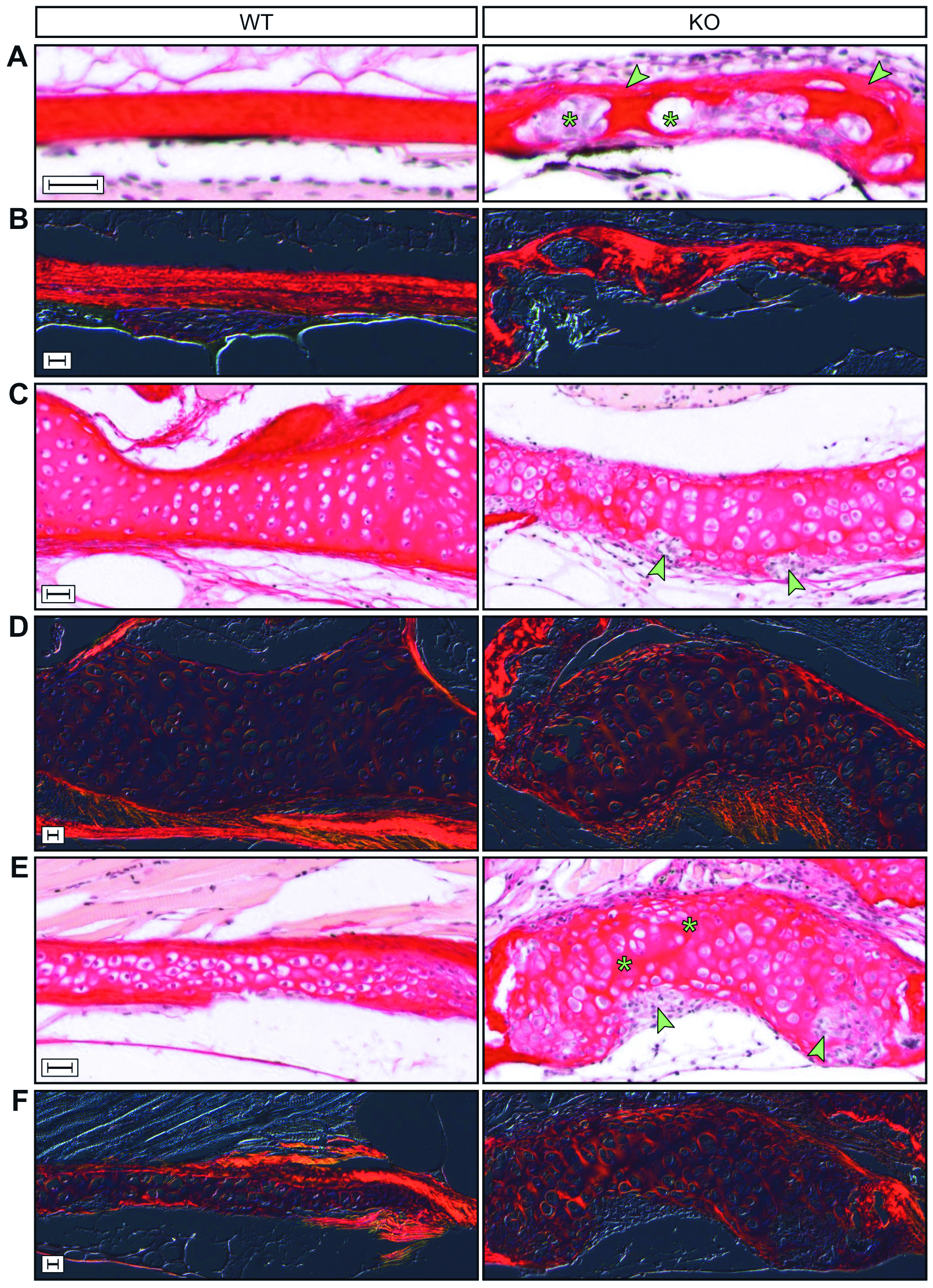

Supplement: Supplementary Data [file ddy168_supp.zip › Fig S7_Revision_HMG.tif]

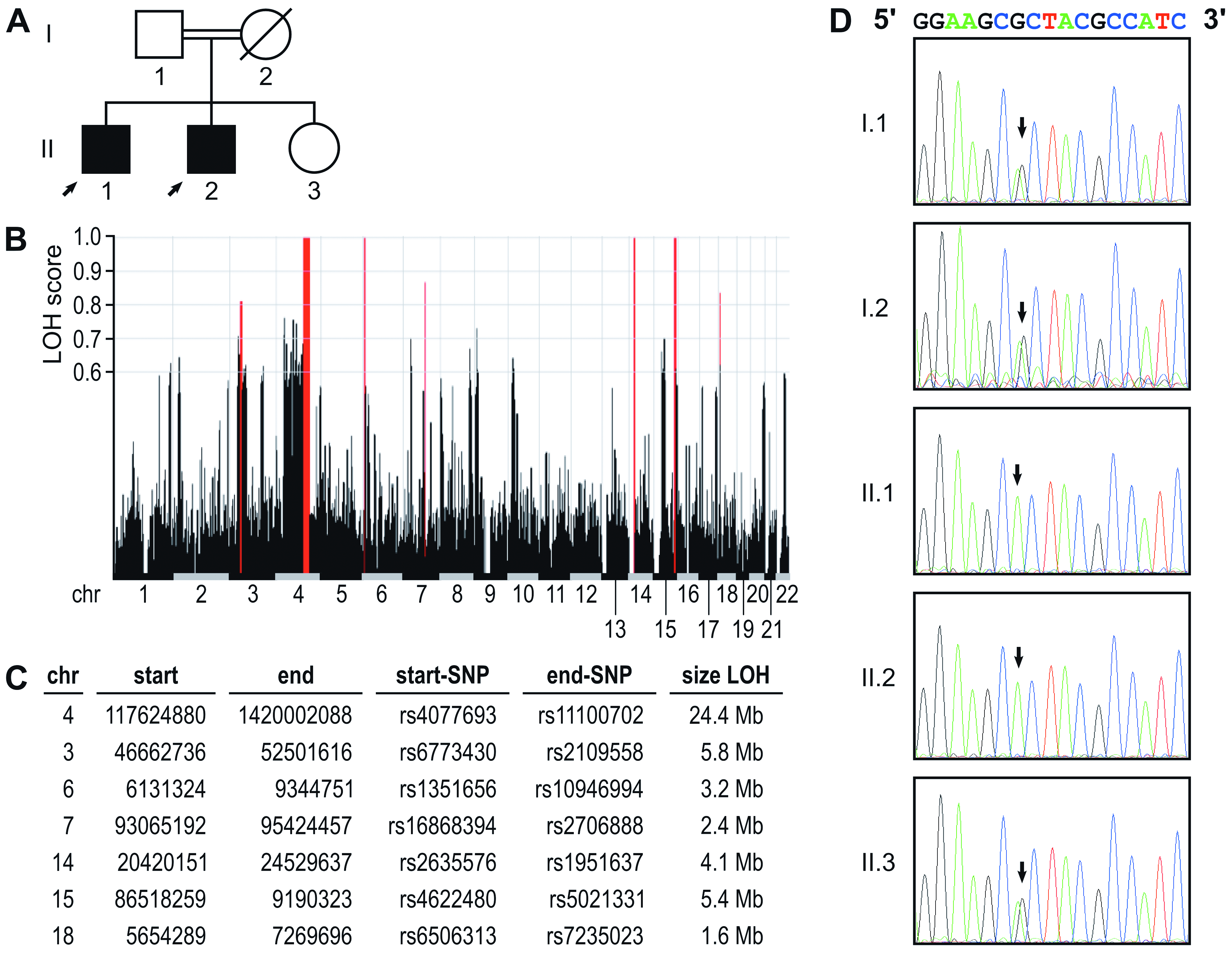

Supplement: Supplementary Data [file ddy168_supp.zip › Fig S1 Revision_HMG.tif]
